# Supplementary material for: Auditive beta stimulation as a countermeasure against driver fatigue
Source: PLoS One. 2021 Jan 11;16(1):e0245251. doi: 10.1371/journal.pone.0245251 (PMC7799808; doi:10.1371/journal.pone.0245251)
Supplement: Attachment — Submitted filename: Response to reviewers.docx [file pone.0245251.s006.docx]

# Response to Reviewers and Editors for the manuscript “Auditive Beta Stimulation as a Countermeasure against Driver Fatigue”

Cologne, 2020, November 28^th^

Dear Editors, dear Reviewers,

In the following we would like to respond to your remarks and suggestions to our study report.

We have checked the style requirements for PLOS One and adjusted the formatting and file naming. We have also updated the Financial disclosure section according to the role of the funders. The funding organizations did not play a role in the study design, data collection and analysis, decision to publish, or preparation of the manuscript and only provided financial support in the form of authors' salaries and. research materials. We have therefore updated the Funding Statement and the Competing Interests Statement:

Amended Financial Disclosure Statement: “This research was supported by the Groupe Renault1 (<https://group.renault.com>) and Infrasonics GmbH2 (<https://www.infrasonics.de>). The support was provided in the form of salary1 (MM, RS, MM) and provision of research equipment1&2. The funders had no role in study design, data collection and analysis, decision to publish, or preparation of the manuscript.”

Amended Competing Interest Statement: “The authors have read the journal's policy and have the following conflicts: This research was supported by the Groupe Renault and Infrasonics GmbH. The authors have no further relevant declarations relating to employment, consultancy, patents, products in developments or marketed products. These sources of funding do not alter the authors' adherence to all the PLoS ONE policies on sharing data and materials.”

Table 1 is now referred in the text of the manuscript to enable the table to be linked. We have included captions for the Supporting Information (SPPS Files) and renamed the files accordingly.

We thank the reviewers for their comments and respond as follows:

Reviewer #1: The discussion part was further elaborated and divided into a discussion part and a conclusion. At the suggestion of the reviewer, we have included further references on the relationship between speed and road safety.

Reviewer #2: The literature section was shortened and revised further.

We hope that the presentation of our study results now meets the requirements and look forward to being informed about the publication soon.

Best Regards,

Markus Mühlensiep

On behalf of all authors
